# Combined effect of SAR-endolysin LysKpV475 with polymyxin B and Salmonella bacteriophage phSE-5

**DOI:** 10.1099/mic.0.001462

**Published:** 2024-05-13

**Authors:** Marco Gontijo, Mateus Pereira Teles, Hugo Martins Correia, Genesy Pérez Jorge, Isabella Carolina Rodrigues Santos Goes, Anthony Jhoao Fasabi Flores, Márcia Braz, Lucas de Moraes Ceseti, Priscila Zonzini Ramos, Ivan Rosa e Silva, Pedro Marcus Pereira Vidigal, Jörg Kobarg, Rafael Miguez Couñago, Cristina Elisa Alvarez-Martinez, Carla Pereira, Carmen S. R. Freire, Adelaide Almeida, Marcelo Brocchi

**Affiliations:** 1 Departamento de Genética, Evolução, Microbiologia e Imunologia, Instituto de Biologia, Universidade Estadual de Campinas (UNICAMP), Campinas, SP 13083-862, Brazil; 2 Laboratório Nacional de Biociências (LNBio), Centro Nacional de Pesquisa em Energia e Materiais (CNPEM), Campinas, SP 13083-970, Brazil; 3 Department of Biology, and Centre for Environmental and Marine Studies (CESAM), University of Aveiro, Aveiro, Portugal; 4 Research Group Statistics and Mathematical Modeling Applied to Educational Quality (GEMMA), University of Sucre, Sincelejo, Sucre, Colombia; 5 Centro de Química Medicinal, Centro de Biologia Molecular e Engenharia Genética, Universidade Estadual de Campinas (UNICAMP), Campinas, SP 13083-970, Brazil; 6 Faculdade de Ciências Farmacêuticas, Universidade Estadual de Campinas (UNICAMP), Campinas, SP 13083-871, Brazil; 7 Núcleo de Análise de Biomoléculas (NuBioMol), Universidade Federal de Viçosa (UFV), Viçosa, MG 36570-900, Brazil; 8 CICECO – Aveiro Institute of Materials, Department of Chemistry, University of Aveiro, 3810-193, Aveiro, Portugal

**Keywords:** active packaging, antibiotic substitute, antimicrobial peptide, genome mining, phage-derived lytic enzymes

## Abstract

Endolysins are bacteriophage (or phage)-encoded enzymes that catalyse the peptidoglycan breakdown in the bacterial cell wall. The exogenous action of recombinant phage endolysins against Gram-positive organisms has been extensively studied. However, the outer membrane acts as a physical barrier when considering the use of recombinant endolysins to combat Gram-negative bacteria. This study aimed to evaluate the antimicrobial activity of the SAR-endolysin LysKpV475 against Gram-negative bacteria as single or combined therapies, using an outer membrane permeabilizer (polymyxin B) and a phage, free or immobilized in a pullulan matrix. In the first step, the endolysin LysKpV475 in solution, alone and combined with polymyxin B, was tested *in vitro* and *in vivo* against ten Gram-negative bacteria, including highly virulent strains and multidrug-resistant isolates. In the second step, the lyophilized LysKpV475 endolysin was combined with the phage phSE-5 and investigated, free or immobilized in a pullulan matrix, against *Salmonella enterica* subsp. *enterica* serovar Typhimurium ATCC 13311. The bacteriostatic action of purified LysKpV475 varied between 8.125 μg ml^−1^ against *Pseudomonas aeruginosa* ATCC 27853, 16.25 μg ml^−1^ against *S*. *enterica* Typhimurium ATCC 13311, and 32.50 μg ml^−1^ against *Klebsiella pneumoniae* ATCC BAA-2146 and *Enterobacter cloacae* P2224. LysKpV475 showed bactericidal activity only for * P. aeruginosa* ATCC 27853 (32.50 μg ml^−1^) and *P. aeruginosa* P2307 (65.00 μg ml^−1^) at the tested concentrations. The effect of the LysKpV475 combined with polymyxin B increased against *K. pneumoniae* ATCC BAA-2146 [fractional inhibitory concentration index (FICI) 0.34; a value lower than 1.0 indicates an additive/combined effect] and *S*. *enterica* Typhimurium ATCC 13311 (FICI 0.93). A synergistic effect against *S*. *enterica* Typhimurium was also observed when the lyophilized LysKpV475 at ⅔ MIC was combined with the phage phSE-5 (m.o.i. of 100). The lyophilized LysKpV475 immobilized in a pullulan matrix maintained a significant S*almonella* reduction of 2 logs after 6 h of treatment. These results demonstrate the potential of SAR-endolysins, alone or in combination with other treatments, in the free form or immobilized in solid matrices, which paves the way for their application in different areas, such as in biocontrol at the food processing stage, biosanitation of food contact surfaces and biopreservation of processed food in active food packing.

## Introduction

Deaths caused by infections with multidrug-resistant (MDR) bacteria are widespread. The most recent antimicrobial-resistance report shows that among the bacterial species commonly associated with deadly diseases due to multidrug resistance, more than half are Gram-negative bacteria, including *Escherichia coli*, *Klebsiella pneumoniae*, *Acinetobacter baumannii*, *Pseudomonas aeruginosa* and *Salmonella enterica*, accounting for nearly three million deaths worldwide in 2019, which corresponds to about 60 % of the total deaths associated with MDR [1]. MDR bacteria have threatened public health, especially in poor and lower-to-middle-income countries [2,3].

Over the years, the development and approval of new antimicrobials have decreased considerably in parallel with the emergence of MDR bacteria [4]. The search for alternative antimicrobial agents has strengthened, focusing on novel approaches, such as enzybiotics and other antimicrobial proteins [5]. Enzybiotics are a class of proteins with antimicrobial properties, first described based on the enzymatic and antibiotic activities of phage-derived lytic enzymes [6]. These enzymes catalyse the cleavage of peptidoglycan, a bacterial cell wall component, affecting the mechanical resistance to osmotic pressure and leading to cell lysis [7]. While many studies have shown the potential of endolysins against Gram-positive bacteria [8,9], the outer membrane of Gram-negative bacteria hampers the use of endolysins due to the protective layer surrounding the cell wall [7,10].

In this context, the action of endolysins against Gram-negative pathogens has been evaluated for: (i) native endolysins; (ii) combinations of native endolysins and outer membrane permeabilizers; and (iii) engineered endolysins [7]. Previous studies reported that the outer membrane permeation of native endolysins is influenced by polyhistidine tags [11] and transmembrane portions at the N-terminus of endolysin, named the signal-anchor-release (SAR) domain [12]. However, the fundamental role of each of these structures remains inconclusive.

Polymyxin E and polymyxin B are secondary metabolite nonribosomal peptides produced by soil bacteria [13,14]. It has been suggested that polymyxins interact electrostatically with the bacterial outer membrane LPS, displacing the magnesium and calcium ions that stabilize the outer membrane structure. These chemical interactions provoke structural and mechanical alterations in the bacterial outer membrane that destabilize its integrity and allow the insertion of the polymyxin molecule into the membrane [13,15, 16].

Therefore, the main aim of the present study was to evaluate the antimicrobial activity of a native SAR-endolysin, identified through a genomic/metagenomic screening [17,18], against Gram-negative bacteria, including MDR strains. To evaluate the potential applications of this SAR-endolysin, its activity was also assessed in combination with the outer membrane permeabilizer polymyxin B and with a phage, in the free form or immobilized in a pullulan matrix. Pullulan films were selected since pullulan edible coatings and films have been successfully used to prolong the shelf life of food products [19]. Thus, this study proposed an *in vitro* assessment of ‘active’ pullulan films against the important foodborne pathogen *S. enterica* subsp. *enterica* serovar Typhimurium.

## Methods

### Bacterial strains, plasmids and phages

Bacterial strains and plasmids used are summarized in Table 1. The bacterial strains *K. pneumoniae* P1298, *A. baumannii* P3380, *P. aeruginosa* P2307 and *Enterobacter cloacae* P2224 were kindly provided by the Instituto Nacional de Controle de Qualidade em Saúde (INCQS), a subdivision of the Fundação Oswaldo Cruz (Fiocruz). The *S*. *enterica* Typhimurium SL1344 was kindly given by Professor Roy Curtiss III (Department of Infectious Diseases and Immunology, University of Florida, USA). *S*. *enterica* Typhimurium ATCC 13311 and phage phSE-5 were used in the time-kill curve assays. Bacteria were cultivated in Luria–Bertani (LB) broth at 37 °C, 100 r.p.m. or on LB agar plates for 12–18 h. Phage phSE-5 was isolated from the sewage network of Aveiro (Portugal) in previous work [20]. Plasmids were purchased from GenScript.

**Table 1.** Plasmids and bacterial strains used in the study

*E.coli* strain used for protein expression		
**Strain**	**Selection marker**	**Characteristics**
DH5α	None	Mutations in recA1 improve insert stability, and endA enhances the yield and quality of inserted DNA [77].
Lemo21(DE3)	Chloramphenicol	Contains the plasmid pACYC184 harbouring lysY, which encodes a lysozyme, a natural inhibitor of phage T7 RNA polymerase. Thus, it is suitable for expressing toxic and membrane proteins [78].
**Plasmids**		
**Plasmid**	**Selection marker**	**Characteristic**
pET28a	Kanamycin	T7 promoter
pET29b- LysKpV475	Kanamycin	T7 promoter, encodes for LysKpV475 with a 6-His tag
**Strains used in the antimicrobial screening**		
**Strain**	**Isolation source**	
*Klebsiellapneumoniae* P1298	Liquor from a patient with meningitis	
*Klebsiellapneumoniae* ATCC BAA-2146	Urine from a hospitalized patient	
*Acinetobacterbaumannii* P3380	Unknown	
*Acinetobacterbaumannii* ATCC 19606	Urine from a hospitalized patient	
*Pseudomonasaeruginosa* P2307	Pulmonary secretion	
*Pseudomonasaeruginosa* ATCC 27853	Blood	
*Enterobactercloacae* P2224	Immunobiological contaminant	
*Enterobactercloacae* ATCC 35030	Unknown	
*Salmonellaenterica* Typhimurium SL1344	Calf intestine	
*Salmonellaenterica* Typhimurium ATCC 13311	Faeces; food poisoning	
*Staphylococcusaureus* ATCC 29213	Hospital patient injury	

### Endolysin LysKpV475

Putative SAR-endolysins identified in the literature [17,18, 21, 22] were previously clustered by using principal component analysis (PCA) [18], and SAR-endolysin LysKpV475 (YP_009280719.1), encoded by the *Klebsiella* phage vB_KpnP_KpV475 (NC_031025.1), was selected to pursue the antimicrobial studies. The enzyme belongs to the glycoside hydrolase family 24 (GH24), the most representative of the families in the PCA groups (*n*=123; 87.86 %).

### Phylogenetic analysis of LysKpV475

We performed a phylogeny analysis of LysKpV475 inside the GH24 muramidase superfamily to track its evolutionary relationship among GH24 SAR-endolysins. The sequence for LysPMBT3 (UniProtKB: A0A2I6PHU80), from glycoside hydrolase family 19 (GH19), a SAR-endolysin from the glucosaminidase superfamily, was used as an outgroup to root the phylogenetic tree. The phylogenetic tree was reconstructed using MetaLogo version 1.1.3 [23], as previously described [24]. Multiple sequence alignment (MSA) was determined by mafft version 7.505 [25] and blast version 1.0 [26]. The sequences of all SAR-endolysins used are available in Supplementary File 1 (available via FigShare: https://doi.org/10.6084/m9.figshare.25647966.v1).

### *In silico* structure analysis of SAR-endolysin LysKpV475

Gontijo *et al*. (2022) [18] previously demonstrated that the *in silico* structure of SAR-endolysin LysKpV475 (UniProtKB: A0A1B0Z137) shares structural similarities (*P* value 1.67e−11) with SAR-endolysin R^21^ (UniProtKB: P27359), whose 3D structure was determined by X-ray crystallography [27,28]. Using RoseTTAFold version 3.13 modelling [29], the 3D *in silico* structures of LysKpV475 (UniProtKB: A0A1B0Z137), LysZZ1 (UniProtKB: I3WVU6) as a non-SAR GH24 control, and a LysKpV475 hybrid containing the N-terminus of LysZZ1 were determined. The *in silico* structures were superimposed using fatcat version 2.0 [30] and the structural alignments were visualized using Mol* Viewer version 3.0.2 [31]. Surface-area calculations were performed using the APBS Electrostatics plugin [32] for the PyMOL Molecular Graphics System, version 2.0 (Schrödinger).

### Antibiotic-susceptibility testing

The Kirby–Bauer disc diffusion test was used according to Clinical and Laboratory Standards Institute (CLSI) instructions [33]. The antimicrobial activity of selected antibiotics was tested against *K. pneumoniae* (P1298 and ATCC BAA-2146), *A. baumannii* (P3380 and ATCC 19606), *P. aeruginosa* (P2307 and ATCC 27853), *Enterobacter cloacae* (P2224 and ATCC 35030) and *S*. *enterica* Typhimurium (SL1344 and ATCC 13311). The selection of antimicrobial discs (Oxoid) followed the CLSI guidelines [29]. The bacterial strains were tested for susceptibility using susceptibility discs from Thermo Scientific Oxoid containing cefazolin, cefoxitin, ciprofloxacin, ceftazidime, ceftriaxone, cefotaxime, amikacin, gentamicin, ertapenem, imipenem, meropenem, aztreonam, amoxicillin–clavulanate, ampicillin, trimethoprim–sulfamethoxazole and tetracycline. Antibiotic classes are shown in Tables 2 and S1 (available with the online version of this article). Susceptibility or non-susceptibility was evaluated after incubation at 37 °C for 18 h on Mueller–Hinton agar (Neogem). The diameters of inhibition zones were measured, and bacterial strains were classified as susceptible (S), intermediate (I) and non-susceptible (NS). Multidrug resistance was attributed to strains non-susceptible to at least one antibiotic from three or more antimicrobial classes [34]. Three independent assays were done.

**Table 2.** Antibiotic-resistance profiles of the Gram-negative bacteria evaluated in this study S, Susceptible; R, resistant; I, intermediate resistance; nt, not tested. Inhibition zone diameters (mm) and the respective standard deviation from three biological replicas are shown in Table S1.

Antibiotic	Class	*Klebsiella pneumoniae* P1298	*Klebsiella pneumoniae* ATCC BAA-2146	*Acinetobacter baumannii* P3380	*Acinetobacter baumannii* ATCC 19606	*Pseudomonas aeruginosa* P2307	*Pseudomonas aeruginosa* ATCC 27853	*Enterobacter cloacae* P2224	*Enterobacter cloacae* ATCC 35030	*Salmonella enterica* Typhimurium SL1344	*Salmonella enterica* Typhimurium ATCC 13311
Cefazolin (30 µg)	First cephalosporins	I	R	nt	nt	nt	nt	R	R	I	S
Cefoxitin (30 µg)	Second cephalosporins	S	R	nt	nt	nt	nt	I	R	S	S
Ciprofloxacin (5 µg)	Second fluoroquinolones	I	R	S	S	S	S	I	S	S	S
Ceftazidime (30 µg)	Third cephalosporins	S	R	S	R	S	S	S	R	S	S
Ceftriaxone (30 µg)	Third cephalosporins	I	R	I	R	nt	nt	R	R	I	S
Cefotaxime (30 µg)	Third cephalosporins	I	R	S	R	nt	nt	I	R	I	S
Amikacin (30 µg)	Aminoglycosides	S	R	I	S	S	S	S	S	S	R
Gentamicin (10 µg)	Aminoglycosides	S	R	I	S	S	S	I	S	S	S
Ertapenem (10 µg)	Carbapenems	S	R	nt	nt	nt	nt	S	I	S	S
Imipenem (10 µg)	Carbapenems	I	R	S	S	S	S	I	S	S	S
Meropenem (30 µg)	Carbapenems	I	R	S	S	S	S	I	S	S	S
Aztreonam (30 µg)	Monobactams	S	R	nt	nt	I	S	S	R	S	R
Amoxicillin–clavulanate (30 µg)	Penicillins	R	R	nt	nt	nt	nt	R	R	S	S
Ampicillin (10 µg)	Penicillins	R	R	nt	nt	nt	nt	R	R	R	R
Trimethoprim–sulfamethoxazole (30 µg)	Sulfonamides	nt	nt	S	R	nt	nt	nt	nt	nt	nt
Tetracycline (30 µg)	Tetracyclines	S	R	nt	nt	nt	nt	R	S	S	R

### *Galleria mellonella* larvae survival after infection

The *G. mellonella* larvae infection model has been widely used as a preliminary infectious disease model due to its ease of use and the similarities of its innate immune system to vertebrates [35,36]. Therefore, to assess the virulence of the bacterial isolates and to verify the antimicrobial action of the proposed SAR-endolysins against virulent bacteria, we used the *G. mellonella* infection model.

This assay was performed as previously described [37], and was used to select the MDR and virulent bacterial isolates and to evaluate the LysKpV475 activity against the selected isolates. Groups of 10 larvae (approximately 200–250 mg) were injected with 10 µl each bacterium suspension, ranging from 10^0^ to 10^7^ c.f.u. per larva, and incubated at 37 °C. PBS was used as the negative control. Then, the larvae survival was scored every 24 h for 96 h, and they were considered dead if they were inert and melanized. Given the higher virulence of *P. aeruginosa* in the *G. mellonella* model, we repeated the experiment and assessed the mortality in smaller intervals to capture the lethal dose (LD_50_). For *P. aeruginosa* strains, the larvae were scored after 12 h and followed every 2 h until 24 h post-injection. Survival curves were plotted and analysed. Three independent assays were done.

### Recombinant expression and purification of endolysin LysKpV475

This experiment was performed as described elsewhere [12,38]. LysKpV475 was cloned with a His6 polyhistidine tag in the C-terminus into the pET29b expression vector. The LysKpV475 synthetic gene in a pET-29b plasmid (GenScript)was used to transform electrocompetent *E. coli* DH5α and Lemo21(DE3)by electroporation (1.8 kV, 25 µF, 200 Ω) (Gene Pulser Xcell Total System; Bio-Rad). For recombinant endolysin expression, cells were cultivated at 37 °C and 150 r.p.m. (Innova) in LB up to an OD_600_ of 0.6. The expression of LysKpV475 was induced by adding 0.1 mM IPTG (Sigma-Aldrich), followed by incubation at 16 °C and 150 r.p.m. for 16 h.

For the purification of LysKpV475, the culture was centrifuged (Beckman J2-21M/E) at 10 000 ***g*** for 30 min. Cells were suspended (1 : 25) in lysis buffer (20 mM NaH_2_PO_4_, 0.5 M NaCl 0.5, 1 % w/v glycerol, pH 7.4) and lysed by sonication (Cole-Parmer; 10 cycles with 30 s pulse and 30 s pause). Cell debris was removed by centrifugation at 10 000 ***g*** for 30 min at 4 °C (Eppendorf 5810R), and the supernatant was filtered (0.22 µm) and applied to Ni^2+^ NTA resin on HisTrap columns (Zymo Research). Elution fractions were analysed using standard denaturing 12 % SDS-PAGE gel.

A high-volume purification protocol was designed to increase protein expression yield, purification efficiency and scale. Briefly, cells were cultivated at 37 °C and 150 r.p.m. (Innova) in terrific broth (TB) containing antibiotics (25 μg chloramphenicol ml^−1^ and 50 μg kanamycin ml^−1^) up to an OD_600_ of 0.7. Protein expression was induced with 0.1 mM IPTG for 16 h. For the purification of endolysin LysKpV475, the culture was centrifuged at 10 000*** g*** for 30 min at 4 °C (Beckman Avanti J-26S), and the cells suspended in 2× lysis buffer (100 mM HEPES, pH 7.5, 1.0 M NaCl, 20 % (v/v) glycerol, 20 mM imidazole, 1 mM TCEP – 1 ml (g cell pellet)^−1^]. After suspension, three volumes of the same 1× buffer [3 ml (g cell pellet)^−1^] were added. The cells were lysed by sonication on ice for 10 min using the Sonics Vibra Cell VCX750 ultrasonic cell disrupter (Sonics and Materials) (5 s on, 10 s off; amplitude=35 %). Polyethyleneimine (PEI) was added to the cell lysate to a final concentration of 0.15 %. Cell debris was removed by centrifugation (40 000 ***g*** for 45 min at 4 °C), and supernatant was passed through a 0.22 µm filter before protein purification by immobilized metal ion affinity chromatography (IMAC). The pre-made columns, HisTrap FF (Cytiva), contained 5 ml Ni-Sepharose resin and were equilibrated with three column volumes (CVs) of elution buffer (binding buffer supplemented with 300 mM imidazole – binding buffer is 50 mM HEPES, pH 7.5, 10 % (v/v) glycerol, 10 mM imidazole, 1 mM TCEP) and 5 CVs of binding buffer. Fractions for the flow-through, 10 mM imidazole wash (in binding buffer, 10 CVs), 30 mM imidazole wash (in binding buffer, 5 CVs) and 300 mM imidazole elution (in binding buffer, 3 CVs) were collected and analysed by 12 % SDS-PAGE gel. The proteins were pooled together for the final purification step – size exclusion chromatography (SEC) – and then loaded onto a pre-equilibrated Hiload 16/600 Superdex 200 pg (Sigma-Aldrich) in gel filtration buffer (20 mM HEPES, pH 7.5, 0.5 M NaCl, 10 % (v/v) glycerol, 0.5 mM TCEP). IMAC and SEC were performed in the AKTApure system (GE Healthcare). The protein purification profile was assessed by UV absorption spectroscopy and 12 % SDS-PAGE gel.

### Endolysin LysKpV475 susceptibility testing

The MIC and minimum bactericidal concentration (MBC) for endolysin LysKpV475 were determined in triplicate using the microdilution method according to CLSI guidelines [39]. The antimicrobial effect of the endolysin LysKpV475 was tested with and without polymyxin B (Sigma Aldrich) against *Staphylococcus aureus* (ATCC 29213), *K. pneumoniae* (P1298 and ATCC BAA-2146), *A. baumannii* (P3380 and ATCC 19606), *P. aeruginosa* (P2307 and ATCC 27853), *Enterobacter cloacae* (P2224 and ATCC 35030) and *S*. *enterica* Typhimurium (SL1344 and ATCC 13311). A density of 0.5 MacFarland scale was obtained by the bacterial growth (OD=0.6); dilution 1 : 100 in 0.85 % saline solution. The endolysin concentrations ranged from 6.8×10^−3^ to 28 µg ml^−1^, and the polymyxin B concentration ranged from 1 to 2.4×10^−4^ µg ml^−1^. His-elution buffer (Zymo Research), containing imidazole, was used as a negative control. A positive control of bacteria culture without antimicrobials was also included. The MIC was defined as the lowest concentration that showed no growth in the Mueller–Hinton broth (Liofilchem). Three independent assays were done.

The greatest bottleneck for endolysin activity against Gram-negative bacteria is the low permeability of the outer membrane [7]. Therefore, a microdilution assay was performed to measure antibiotic interactions [40] to define the fractional inhibitory concentration index (FICI) and the checkerboard assay to quantify possible synergistic or antagonist interactions [41] between LysKpV475 and polymyxin B. The FICI was calculated for *K. pneumoniae* ATCC BAA-2146 and *S*. *enterica* Typhimurium ATCC 13311 as model strains.

Given the relevance of *S*. *enterica* Typhimurium in the food industry, the purified endolysin LysKpV475 was lyophilized, referred to as lyophilized from now on, and further tested against *S*. *enterica* Typhimurium ATCC 13311, as explained in the following sections.

### Lyophilization of purified LysKpV475

The protein aliquots were frozen in liquid nitrogen (−196 °C) in freeze-drying flasks, forming a thin layer to facilitate water sublimation. The aliquots were then dried in a vacuum sublimation lyophilizer (Micromodulyo 115; Thermo Electron Corporation) for 48 h and stored in a freezer at −20 °C.

### Time-kill curve assays with endolysin LysKpV475, phage phSE-5, and a combination of endolysin and phage

Previous studies have shown that phage phSE-5 at an m.o.i. of 100 is effective against *S*. *enterica* Typhimurium ATCC 13311 and results in a frequency of *S*. *enterica* Typhimurium spontaneous phage-resistant mutants of 1.27×10^−4^, similar to a phage cocktail containing three *S*. *enterica* Typhimurium phages [20]. This study aimed to evaluate the effect of a bacteriophage/endolysin cocktail in inhibiting *S*. *enterica* Typhimurium survival and regrowth.

Bacterial inactivation was determined using endolysin LysKpV475, phage phSE-5 at m.o.i. 100, and endolysin LysKpV475 and phage phSE-5 in 96-well microplates. The assays were done in tryptic soy broth (TSB) medium (Liofilchem) with the bacterium *S*. *enterica* Typhimurium ATCC 13311 (final concentration of 10^5^ c.f.u. ml^−1^) with only phage phSE-5 (m.o.i. of 100) (bacteria+phage), with only LysKpV475 (MIC=300 µg ml^−1^) [bacteria+LysKpV475 (MIC)], and with a combination of both LysKpV475 (⅔ MIC=200 µg ml^−1^) and phage phSE-5 (m.o.i. of 100) [bacteria+LysKpV475 (⅔ MIC)+phage]. For each assay, the bacterial control was only inoculated with bacteria. Controls and test samples were incubated at 37 °C, and aliquots were collected at 0 h and after 4, 8, 12, 24, 36 and 48 h of incubation. Culture turbidity was measured by spectrophotometry using a Multiskan FC microplate photometer (Thermo Fisher Scientific) set at 600 nm. Three independent experiments were performed for each condition.

### Preparation of pullulan films incorporating LysKpV475 and/or phage phSE-5

The potential additive effect of the lyophilized LysKpV475 and *Salmonella* phage phSE-5 to inhibit *S*. *enterica* Typhimurium ATCC 13311 was assessed through a growth curve assay. Pullulan was selected to produce active packaging films containing either lyophilized LysKpV475 or a combination of LysKpV475 and *Salmonella* phage phSE-5 [42,43]. A pullulan solution (6 %, w/v) containing 10% (w/v) glycerol was prepared by mixing all components until homogenization. The following pullulan films were prepared: (i) pullulan +lyophilized LysKpV475 (MIC=300 µg ml^−1^); (ii) pullulan +phage phSE-5 (m.o.i. of 100); and (iii) pullulan +lyophilized LysKpV475 (⅔ MIC=200 µg ml^−1^)+phage phSE-5 (m.o.i. of 100). Subsequently, 1 ml of each solution was added to the wells of 24-well plates, which served as templates, and placed at 25 °C until complete evaporation of the solvent [44]. The resulting films were removed from the moulds to verify their integrity and then returned to the well to proceed with the tests. The antibacterial capacity of produced pullulan films was tested against *S*. *enterica* Typhimurium ATCC 13311. The exponential bacteria cultures (final concentration of 10^5^ c.f.u. ml^−1^) were inoculated in 24-well plates with TSB in contact with the film and incubated at 37 °C. For each assay, one bacterial control was included. The bacterial control was only inoculated with *Salmonella*. Controls and test samples were incubated under exactly the same conditions. Aliquots were collected at 0 h and after 4, 8, 12, 24, 36 and 48 h of incubation. Bacterial concentration was determined in triplicate in tryptic soy agar (TSA) medium (Liofilchem) through the drop plate method after an incubation period of 24 h at 37 °C. Three independent experiments were performed for each condition.

### Statistical analysis

A one-way ANOVA test was used to compare the experimental groups, setting the statistical significance at *P*<0.05. GraphPad Prism version 9.5.0 software, licensed by Duke University Office of Information Technology, was used for experimental data processing.

## Results

### Characterization of SAR-endolysins

**Fig. 1.**
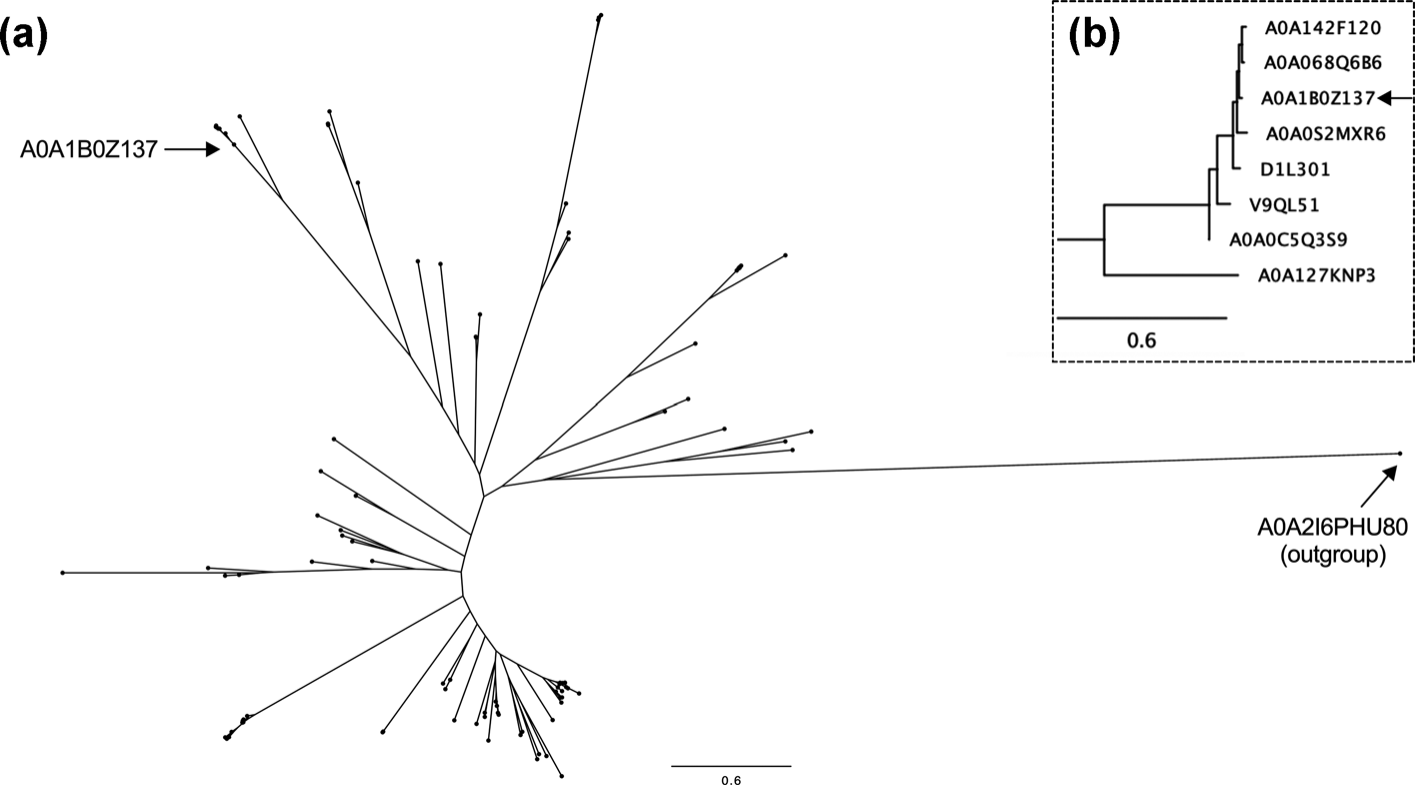
Phylogenetic analysis of all putative SAR-lysozymes (GH24). (**a)** Phylogenetic tree of all GH24 SAR-endolysins described in the literature [18] and LysPMBT3 (UniProtKB: A0A2I6PHU80) from GH19 as an outgroup control. An arrow indicates LysKpV475 (UniProtKB: A0A1B0Z137). (**b)** Highlight of the closest clade to LysKpV475 (UniProtKB: A0A1B0Z137). Bars indicate a phylogenetic distance of 0.6 amino acid substitutions per site. For more detailed information on the SAR-endolysin sequences and clades in the phylogenetic tree, please refer to Supplementary Files 1 and 2, respectively.

The studied SAR-endolysins are closely related and have structural similarities. The phylogenetic tree of SAR-lysozymes (GH24) (Fig. 1a) shows a conserved clade of SAR-lysozymes encoded by *Klebsiella* phages, including LysKpV475 (A0A1B0Z137), which is highlighted in Fig. 1(b). LysKpV475 shares at least 89.45 % sequence identity, *E* value <3e−139, and query coverage >98 % with other closely related SAR-lysozymes (Fig. 1b). However, the sequence identity between LysKpV475 and SAR-lysozyme A0A127KNP3, the closest sequence to this clade, also shown in Fig. 1(b), drops to only 43.72 % identical (query coverage=98 %; *E* value=7e−58). For more detailed information on the clades in the phylogenetic tree, please refer to Supplementary File 2 (available via FigShare: https://doi.org/10.6084/m9.figshare.25647966.v1).

**Fig. 2.**
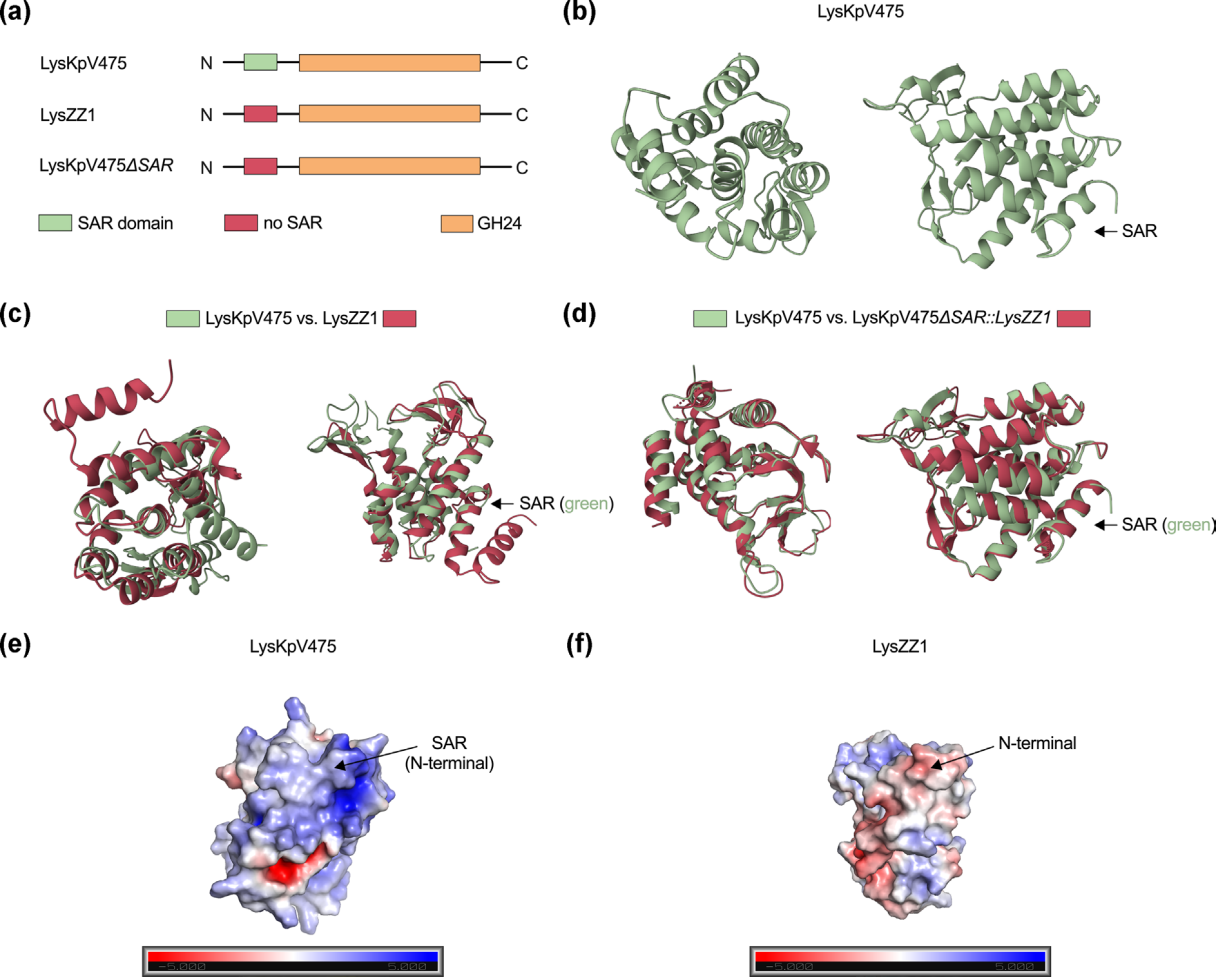
*In silico* structural prediction of LysKpV475 (UniProtKB: A0A1B0Z137), LysZZ1 (UniProtKB: I3WVU6) and LysKpV475ΔSAR::LysZZ1. (a) Overview of protein versions used for *in silico* analysis. The N-terminal portion of the SAR domain of LysKpV475 was replaced with the N-terminus of LysZZ1 (LysKpV475ΔSAR::LysZZ1), an endolysin that does not have a SAR domain. (b) *In silico* prediction structure of LysKpV475. (c) Superimposed structures of LysKpV475 and LysZZ1. (d) Superimposed structures of LysKpV475 and LysKpV475ΔSAR::LysZZ1. The arrow indicates the SAR domain. (e) Surface electrostatic potential of the LysKpV475 *in silico* structure. (f) Surface electrostatic potential of the LysZZ1 *in silico* structure. The electrostatic potential in (e) and (f) ranges from −5.00 kTe-1 (negatively charged; shown in red) to +5.00 kTe-1 (positively charged; shown in blue), where k denotes the Boltzmann constant and e represents the electron charge.

To investigate whether the SAR domain has a distinctive structure that could affect outer membrane permeability, *in silico* structure prediction and pairwise structural analysis were used to compare LysKpV475, a SAR-lysozyme, and LysZZ1, a canonical lysozyme (Fig. 2a, b). The superimposed structures of LysKpV475 and LysZZ1 indicate that the N-terminus of SAR-lysozymes and canonical lysozymes share similar secondary structures, characterized by an α-helix (*P* value 1.04e−10) (Figs 2c and S1A). However, LysZZ1 has an additional α-helix at the N-terminus compared to LysKpV475. By performing an *in silico* mutation to replace the N-terminus of LysKpV475 with that of LysZZ1 (Fig. 2a), it was observed that the model 3D structures were more structurally similar (*P* value 0.00e+00; Fig. S1B) at the N-terminal portion (Fig. 2d). Furthermore, the SAR-domain has a net-positive charge (Fig. 2e). In contrast, the N-terminal portion of canonical lysozymes has a net-negative charge (Fig. 2f).

### Antibiotic-susceptibility testing and *G. mellonella* survival assay

**Fig. 3.**
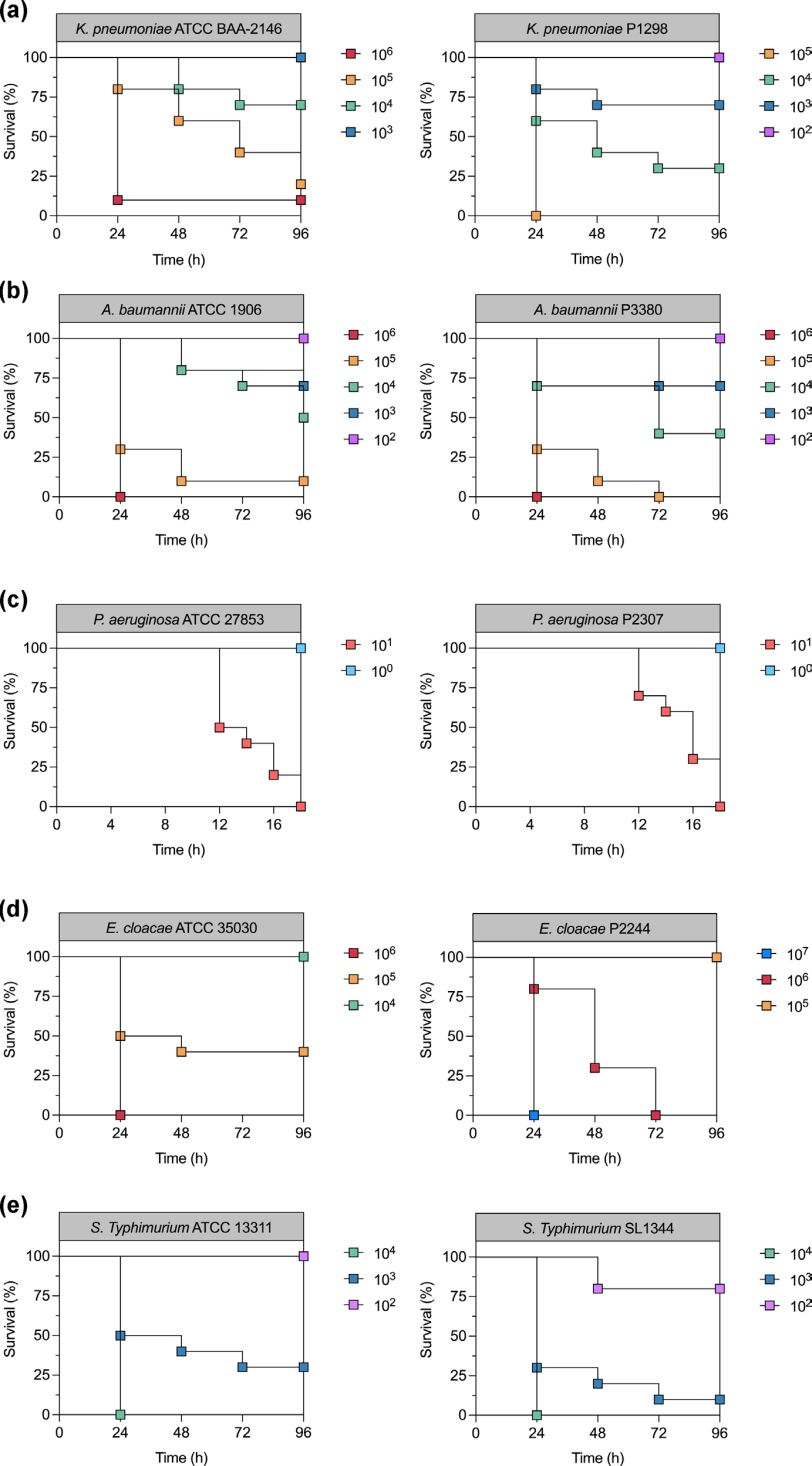
*G. mellonella* Kaplan–Meier survival curves obtained for the bacterial strains: (**a)** *K. pneumoniae* ATCC BAA-2146 and P1298; (**b)** *A. baumannii* ATCC 19606 and P3380; (**c)** *P. aeruginosa* ATCC 27853 and P2307; (**d)** *Enterobacter cloacae* ATCC 35030 and P2224; (**e)** *S*. *enterica* Typhimurium ATCC 13311 and SL1344.

To select MDR Gram-negative bacteria for the analysis of endolysin activity, an antibiotic-susceptibility testing as standardized by CLSI was performed [33], and the results are displayed in Tables 2 and S1. Among the isolates evaluated in this study, *K. pneumoniae* ATCC BAA-2146, *Enterobacter cloacae* ATCC 35030, *Enterobacter cloacae* P2224 and *S*. *enterica* Typhimurium ATCC 13311 showed resistance to more than three classes of antibiotics and were classified as MDR. Additionally, the virulence of the MDR strains was first assessed by the *G. mellonella* killing assay as a model to evaluate the virulence of the strains (Fig. 3). The most virulent strains in *G. mellonella* were *P. aeruginosa* strains P2307 and ATCC 27853 (Table 3, Fig. 3).

**Table 3.** Calculated LD_50_ of bacterial strains in *G. mellonella* *G. mellonella* Kaplan–Meier survival curves are shown in Fig. 3.

Strain	LD_50_ (c.f.u. per larva)
*Klebsiella pneumoniae* P1298	3.23×10^3^
*Klebsiella pneumoniae* ATCC BAA-2146	4.27×10^4^
*Acinetobacter baumannii* P3308	9.70×10^3^
*Acinetobacter baumannii* ATCC 1906	1.53×10^4^
*Pseudomonas aeruginosa* P2307	5.38×10^0^
*Pseudomonas aeruginosa* ATCC 27593	3.79×10^0^
*Enterobacter cloacae* P2244	9.56×10^5^
*Enterobacter cloacae* ATCC 35030	3.01×10^5^
*Salmonella enterica* Typhimurium SL1344	1.17×10^3^
*Salmonella enterica* Typhimurium ATCC 13311	1.01×10^4^

### Endolysin LysKpV475 purification and susceptibility testing

**Fig. 4.**
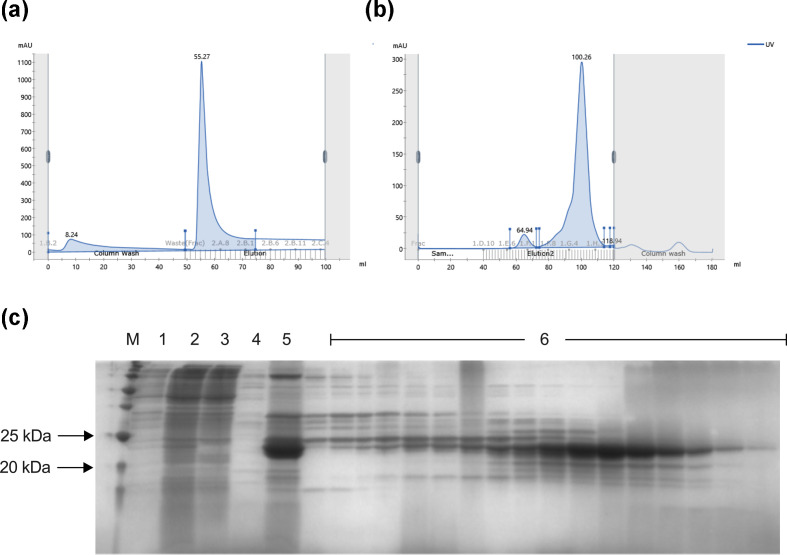
(a, b) UV absorption spectroscopy used to assess the purity of LysKpV475. (**a)** Chromatogram for lysate purification by IMAC with elution using an imidazole gradient (0–300 mM). (**b)** Chromatogram for isocratic purification after IMAC by SEC. Both by ÄKTA pure equipment. (**c)** SDS-PAGE containing the molecular mass marker (M), an aliquot of total cell lysate after sonication (1), an aliquot of soluble supernatant after centrifugation (2), an aliquot of the fraction retained in the metal affinity column (IMAC FT) (3), an aliquot of the wash solution from the metal affinity column (IMAC wash) (4), an aliquot of the elution from the affinity column (IMAC elution) (5) and aliquots of fractions obtained by SEC (6).

Protein expression was assessed by SDS-PAGE gels after induction with 0.1, 0.4 and 1.0 mM IPTG (Fig. S2A) and similar band intensities were observed with all IPTG concentrations (Fig. S2B). The following purifications were conducted using the lowest IPTG concentration (0.1 mM). In addition, the bacterial strains *E. coli* Lemo21(DE3), which contains the pLemo plasmid, *E. coli* Lemo21(DE3) +pET28 a, and *E. coli* +pET29bLysKpV475, were used (Fig. S3A). Signs of protein expression were observed 4 h after induction (Fig. S3B, C), and the differences in bacterial growth between the induced and non-induced systems were more pronounced 16 h after induction (Fig. S3B, D). The protein purification profile was also assessed by UV absorption spectroscopy (Fig. 4a, b) and 12 % SDS-PAGE gel (Fig. 4c).

To test the hypothesis that SAR-lysozymes have antimicrobial activity against the selected Gram-negative bacteria, a broth microdilution assay adapted from the CLSI method [39] was performed, and the results are summarized in Table 4. The MIC, referencing a bacteriostatic action of LysKpV475, varied between 8.125 μg ml^−1^ for *P. aeruginosa* ATCC 27853, and 32.50 μg ml^−1^ for *K. pneumoniae* ATCC BAA-2146 and *Enterobacter cloacae* P2224. The MBC, representing the bactericidal action, was observed only for *P. aeruginosa* ATCC 27853 (32.50 μg ml^−1^) and *P. aeruginosa* P2307 (65.00 μg ml^−1^). His-elution buffer did not show any antimicrobial activity alone. Still, we cannot rule out an additional effect of the imidazole present in the buffer with the impact of the endolysin LysKpV475.

**Table 4.** MIC and MBC concentrations of purified LysKpV475 against the tested bacteria

Bacteria	Gram	MIC (µg ml^−1^)	MBC (µg ml^−1^)
*Staphylococcus aureus* ATCC 29213	+	16.25	>65.00
*Klebsiella pneumoniae* ATCC BAA-2146	−	32.50	>65.00
*Klebsiella pneumoniae* P1298	−	16.25	>65.00
*Acinetobacter baumannii* ATCC 19606	−	16.25	>65.00
*Acinetobacter baumannii* P3380	−	16.25	>65.00
*Pseudomonas aeruginosa* ATCC 27853	−	8.125	32.50
*Pseudomonas aeruginosa* P2307	−	16.25	65.00
*Enterobacter cloacae* ATCC 35030	−	16.25	>65.00
*Enterobacter cloacae* P2224	−	32.50	>65.00
*Salmonella enterica* Typhimurium ATCC 13311	−	16.25	>65.00
*Salmonella enterica* Typhimurium ATCC 13311	−	300.00^*^	nt
*Salmonella enterica* Typhimurium SL1344	−	16.25	>65.00

1 Lyophilized LysKpV475.nt, Non-tested.

2 *Lyophilized LysKpV475.Non-tested

### Combined effect of LysKpV475 with polymyxin B or *Salmonella* phage phSE-5

The results obtained from the checkerboard assay (Fig. S4, Table S2), shown in (Table 5), demonstrate that LysKpV475 and polymyxin B had an additive/combined effect promoting bactericidal activity against *K. pneumoniae* ATCC BAA-2146 (FICI=0.340) and *S*. *enterica* Typhimurium ATCC 13311 (FICI=0.930), since FICI values lower than 1.0 indicate an additive/combined effect [41].

**Table 5.** MICs and MBCs of purified LysKpV475 and polymyxin B alone or combined against *K. pneumoniae* ATCC BAA-2146 and *S. enterica* ATCC 13311. FICI values were used to assess antimicrobial synergism.

Bacteria	Alone		Combination		FICI
	MIC (µg ml^-1^)				
	Polymyxin B	LysKpV475	Polymyxin B	LysKpV475	
*Klebsiellapneumoniae* ATCC BAA-2146	0.5	16.25	0.5	14	1.86
*Salmonella* Typhimurium ATCC 13311	0.5	16.25	0.5	14	1.86
	MBC (µg ml^-1^)				
	Polymyxin B	LysKpV475	Polymyxin B	LysKpV475	
*Klebsiellapneumoniae* ATCC BAA-2146	4	>65.00	0.5	14	0.34
*Salmonella* Typhimurium ATCC 13311	2	>65.00	1	28	0.93

After lyophilization, the MIC had an 18-fold increase (Table 4), indicating a decrease in the antimicrobial activity of LysKpV475 when lyophilized. Lyophilized LysKpV475 MBC was not tested. Therefore, the following analyses were conducted using *S*. *enterica* Typhimurium ATCC 13311 as a model and with the lyophilized powder of LysKpV745 (MIC=300 µg ml^−1^).

**Fig. 5.**
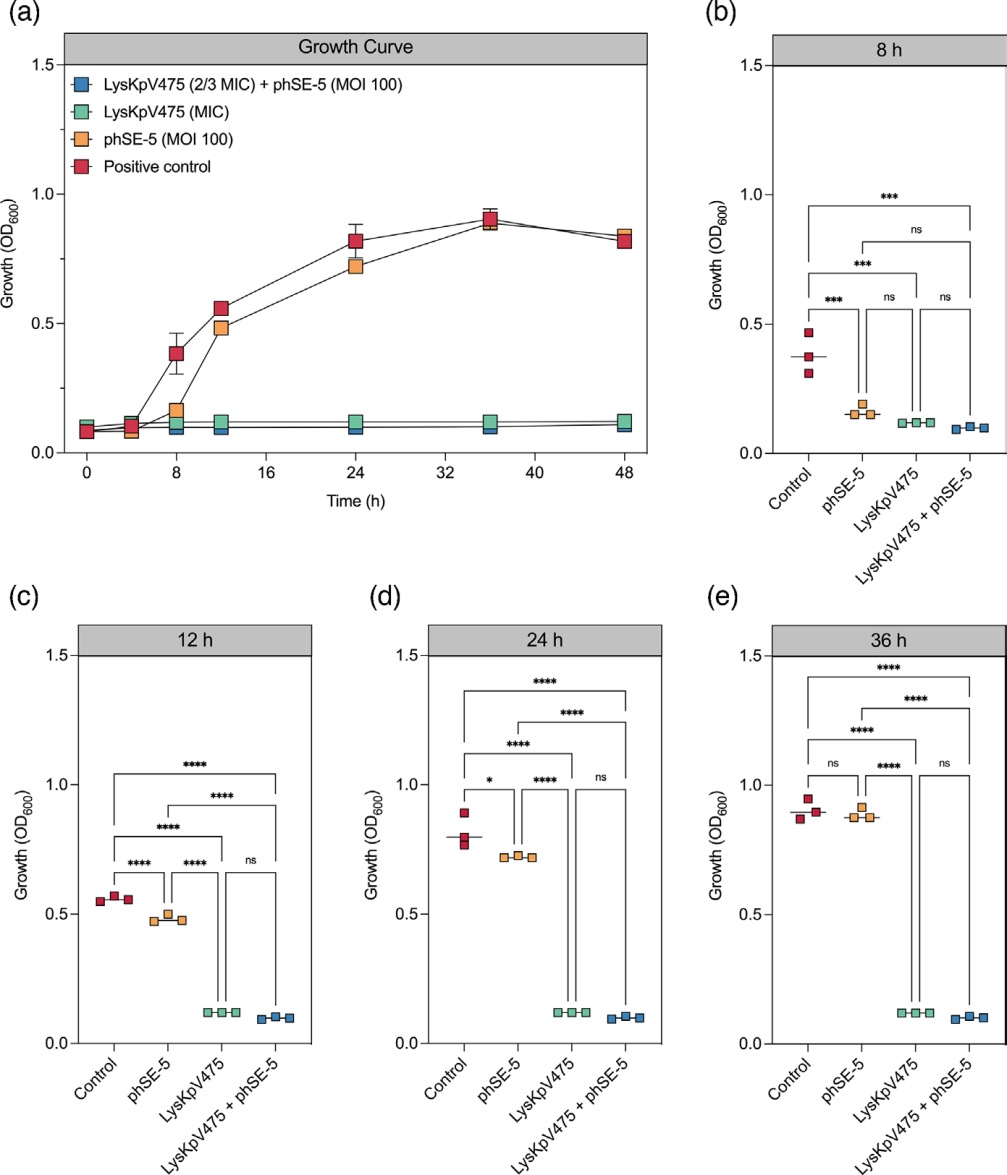
Time-kill curve assay evaluating the synergistic effect *in vitro* of lyophilized LysKpV475 (MIC=300 µg ml^−1^) with phage phSE-5 (m.o.i. 100) against *S*. *enterica* Typhimurium ATCC 13311 (10^5^ c.f.u. ml^-1^). (**a)** Time-kill curve was evaluated for 48 h. (**b–e)** OD_600_ at indicated time points after incubation. Statistical differences were determined by one-way ANOVA and Tukey’s multiple comparisons test (b–e). *, *P*<0.05; ***, *P*<0.001; ****, *P*<0.0001. Differences that are not statistically significant are designated as ns.

Phage phSE-5 can only poorly reduce *S*. *enterica* Typhimurium growth alone (Fig. 5) and can no longer reduce bacterial growth 36 h after incubation (Fig. 5e). However, the addition of LysKpV475 (at MIC) avoids bacterial growth for at least 48 h (Fig. 5a–e). In addition, LysKpV475 at a subinhibitory concentration (at ⅔ MIC) combined with phage phSE-5 inhibits bacterial proliferation similarly to the endolysin (at MIC) treatment, also suggesting a combined effect (Fig. 5a–e).

### Activity of the endolysin LysKpV475 in the pullulan matrix

**Fig. 6.**
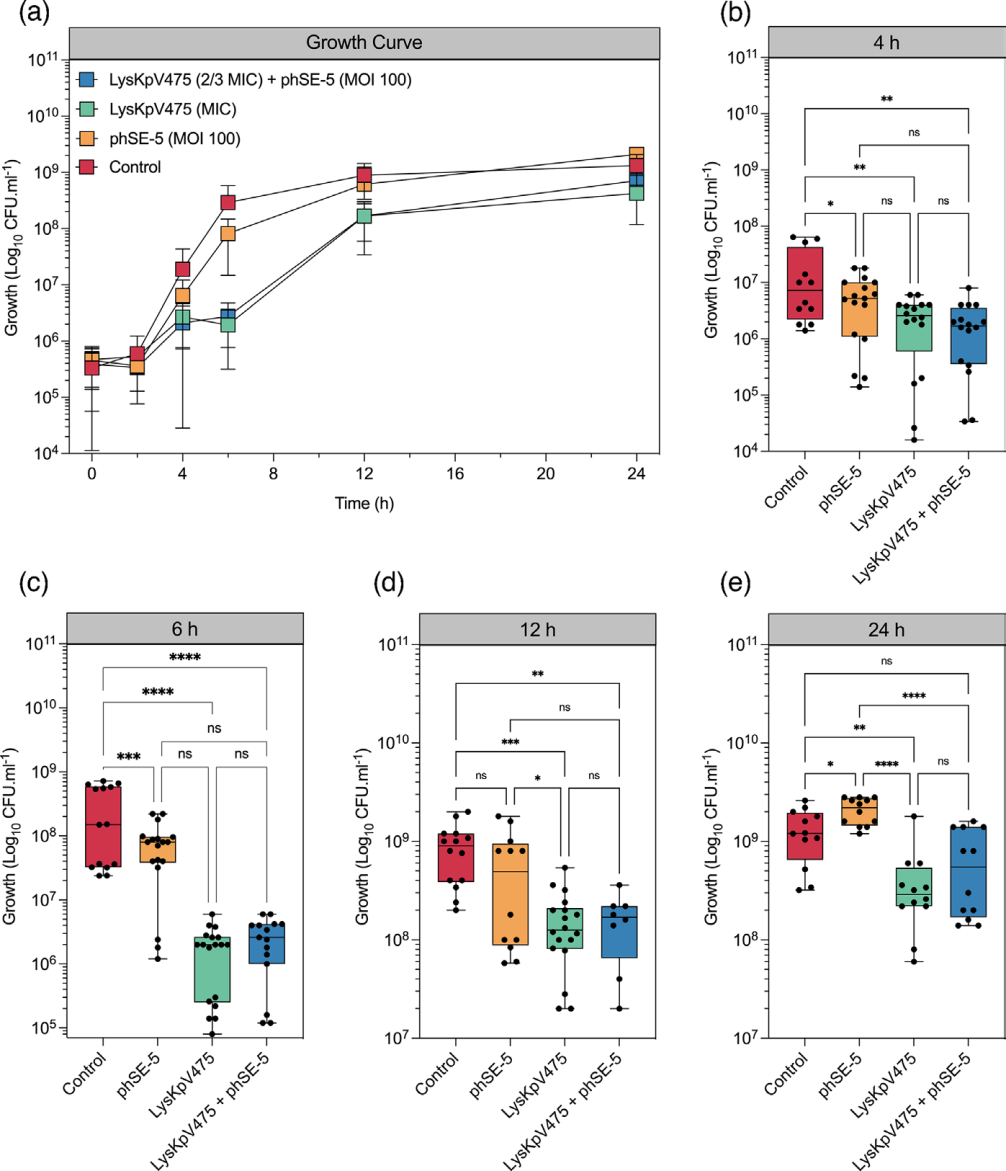
Antimicrobial activity of pullulan films with immobilized antimicrobial lyophilized LysKpV475 and phage phSE-5. (**a)** Time-kill curve assay evaluating the synergistic effect *in vitro* of immobilized lyophilized LysKpV475 (MIC=300 µg ml^−1^) in a pullulan film with phage phSE-5 (m.o.i. 100) against *S*. *enterica* Typhimurium ATCC 13311 (10^5^ c.f.u. ml^−1^). (**b–e) **Growth (c.f.u. ml^−1^) at indicated time points after incubation. Statistical differences were determined by one-way ANOVA and Tukey’s multiple comparisons test (b–e). *, *P*<0.05: **, *P*<0.01: ***, *P*<0.001: ****, *P*<0.0001. Differences that are not statistically significant are designated as ns.

The data in Fig. 6(a, b) show a significant (*P*<0.05) log reduction in the bacterial count after 6 h of incubation in the active packaging model for both LysKpV475 alone, and phage and endolysin at ⅔ MIC. Significant differences in bacterial growth at 4, 6, 12 and 24 h after incubation were also detected (Fig. 6b–e). These data suggest that LysKpV475 (MIC) alone or LysKpV475 (⅔ MIC) combined with phage phSE-5 efficiently reduce bacterial growth after incubation, suggesting their effectiveness when incorporated in an active packaging model.

## Discussion

Numerous studies have confirmed the *in vitro* effectiveness of endolysins to combat multiple Gram-positive pathogens [9]. However, very few studies focused on the *in vitro* application of these enzymes against Gram-negative pathogens due to the limited action of such enzymes against Gram-negative bacteria [7]. Genome sequencing technology and metagenomics have dramatically expanded the understanding of phages and bacterial genomes, allowing screening for virulence, antibiotic-resistance genes [45,46] and antimicrobial peptides [18]. Alternative strategies to combat MDR bacteria include the use of lactic acid bacteria, probiotics and their bacteriocins [47,49], phages [50,53], phage-derived lytic enzymes and other antimicrobial proteins [54,56], as well as plant-based active molecules [57].

Among those strategies, SAR-endolysins are promising candidates for combating infections caused by Gram-negative bacteria due to the hydrophobicity of the SAR domain [17,18]. A previous study by Lim *et al*. [12] evaluated the SAR-endolysin SPN9CC, encoded by the *Salmonella* phage SPN9CC, and observed an active concentration of 300 μg ml^−1^. The authors suggested that the SAR domain could be responsible for penetration into the outer membrane, since endolysin SPN9CC deleted of some amino acids in the N-terminal region did not show lytic activity. However, to date, no other studies have evaluated the exogenous action of SAR-endolysins against Gram-negative bacteria. Thus, we recently proposed an endolysin-screening pipeline to identify SAR-endolysins from genomic and metagenomic data [17,18] and, here, we assessed their potential use against MDR Gram-negative bacteria.

The differences in the overall net charge of the N-terminal portion of endolysins might elucidate the biochemical properties underlying the exogenous action of SAR-endolysins against Gram-negative pathogens. By performing *in silico* mutations and predicting the 3D structure of LysKpV475, the SAR-endolysin evaluated in this study (Fig. 2), it was shown that SAR-endolysins and canonical endolysins belonging to the same lysozyme superfamily share similar secondary structures (Fig. 2c), and that both the structure and biochemistry (Fig. 2d) of the SAR likely play a role in the SAR domain’s interaction with membranes. Bacterial membranes are negatively charged due to electronegative groups in bacterial phospholipids [58]. The overall positive charge of the SAR domain (Fig. 2e), compared to the N-terminal portion of canonical endolysins (Fig. 2f), may favour such interactions with cell membranes. Bacterial LPS, the major component of the bacterial outer membrane, also has a net-negative charge [59,60], pointing to potential SAR-endolysin and LPS interactions. We have also previously shown, by structural analysis, that both protein structure and biochemical composition might play a role in the activity of SAR-endolysins [18]. These results suggest that structural and biochemical interactions likely affect the SAR domain’s interaction with membranes, potentially impacting outer membrane permeability.

*K. pneumoniae* ATCC BAA-2146 has been shown to be resistant to several antibiotics [61,62] and has about 36 chromosomal and plasmid genes associated with antibiotic resistance [61], and *S*. *enterica* Typhimurium ATCC 13311 is a reference bacteria commonly used as a MDR strain [63,64]. To our knowledge, this study is the first report of MDR in *Enterobacter cloacae* ATCC 35030 and P2224. The endolysin LysKpV475, encoded by the *Klebsiella* phage vB_KpnP_KpV475 (NC_031025.1), showed bacteriostatic activity against all the tested Gram-negative bacterial isolates, including MDR strains *Enterobacter cloacae* P2224, *Enterobacter cloacae* ATCC 35030 and *S*. *enterica* Typhimurium ATCC 13311 (Table 4). In addition, LysKpV475 also inhibited *K. pneumoniae* ATCC BAA-2146, an extensively drug-resistant strain (Tables 2 and S1). It is worth noting that bactericidal activity was observed for *P. aeruginosa* ATCC 27853 at 32.50 μg ml^−1^ and *P. aeruginosa* P2307 at 65.00 μg ml^−1^, which were the highly virulent strains against the *G. mellonella* infection model (Fig. 3). In the case of Gram-negative bacteria, the bacteriostatic effect can be desirable since the release of LPS in the body can be avoided [65,66]. These results highlight the potential of this SAR-endolysin against a broad range of MDR and virulent Gram-negative bacteria. The antimicrobial activity of native endolysins against Gram-negative bacteria has rarely been observed [7]. Antonova *et al*. [67] obtained a minimum active concentration of 0.5 μg ml^−1^ for the endolysin LysAm24, LysECD7 and for LysSi3 against *P aeruginosa*, *A. baumannii*, *K. pneumoniae*, *E. coli* and *S*. *enterica* Typhi strains. The authors attributed the outer membrane permeation to the polyhistidine tag with eight amino acids at the C-terminus of the recombinant endolysins. However, additional studies by the same researchers revealed that intrinsic properties other than the His-tag are responsible for such permeation [11]. In addition to these studies, Guo *et al*. [68] observed that the endolysin LysPA26 showed bactericidal activity against *P. aeruginosa* at 500 μg ml^−1^ and Kim *et al*. [69] showed that the recombinant endolysin LysSS had antibacterial activity against *A. baumannii*, *E. coli*, *K. pneumoniae*, *P. aeruginosa* and *Salmonella* with a MIC lower than 750 μg ml^−1^. The SAR-endolysin evaluated in this study, LysKpV475, is active against several Gram-negative bacteria strains (MIC <32.50 µg ml^−1^), including MDR isolates. The results presented in this study further support the claim that SAR-endolysins are native endolysins with exogenous action against Gram-negative bacteria.

Combination of therapies has been an essential ally in the fight against infections caused by MDR pathogens, and has been widely used to prevent and mitigate the risk of MDR-associated diseases [70]. To further assess the antimicrobial activity of LysKpV475, the combined effect of LysKpV475 with polymyxin B, a last-resort antibiotic peptide that disrupts the outer membrane of Gram-negative bacteria [13], was assessed. Despite no synergistic effect on the inhibitory concentration, LysKpV475 with polymyxin B showed a synergistic effect in the lysis of bacterial cells of *K. pneumoniae* ATCC BAA-2146 and *S*. *enterica* Typhimurium ATCC 13311 (Table 4). In addition to polymyxin B, phage phSE-5 also potentiated the efficacy of LysKpV475 (Fig. 5). This synergistic combination of phage and endolysin might be a strategy to prevent bacterial resistance to both phages and endolysins [71,72]. Similar to LysKpV475, which exhibited reduced antibacterial action against *S*. *enterica* Typhimurium ATCC 13311 following lyophilization (Table 4), other proteins [73] and phages are also sensitive to dehydration [52]. However, the lyoprotectants could be a promising solution, as they are hydrophilic compounds that help stabilize and protect the protein during freeze-drying and storage [74]. Lyophilized water-in-oil emulsions have been proposed previously for oral peptide delivery [75]. Further combined studies with a new lyophilized water-in-oil emulsion endolysin must be done in the future.

In order to boost the knowledge transference to the practical, a pullulan-based phage-endolysin film was prepared to simulate the application of the lyophilized LysKpV475 in combination with phage phSE-5 in an antimicrobial food-packaging system [76] (Fig. 6b). Significant reduction in the bacterial counts occurred after 6 h of incubation for LysKpV475 and LysKpV475 combined with phage phSE-5.

### Conclusion

The SAR-endolysin LysKpV475 was expressed and purified, and demonstrated bacteriostatic action against ten Gram-negative pathogens, including MDR and virulent strains. Purified LysKpV475 also exhibited bactericidal action against *P. aeruginosa*. In addition, lyophilized LysKpV475 activity was potentiated by combination with polymyxin B, a polypeptide bactericidal antibiotic targeting bacterial LPS in the outer membrane, and with the *Salmonella* phage phSE-5. These findings underscore the potential of SAR-endolysins as a promising strategy against Gram-negative bacteria, as a single or combined treatment, and the importance of genomics and metagenomics in identifying new antimicrobials. Nevertheless, further studies are necessary to elucidate the role of the SAR domain in the antimicrobial activity of SAR-endolysins, and how to maintain protein stability during lyophilization and immobilization. In addition, further thermal and pH sensitivity assays are required to determine the optimal conditions for LysKpV475 application.

## supplementary material

10.1099/mic.0.001462 Uncited Table S1.

10.1099/mic.0.001462 Uncited Fig. S1.

## Abbreviations

CAPES: Coordenação de Aperfeiçoamento de Pessoal de Nível Superior
CLSI: Clinical and Laboratory Standards Institute
CNPq: Conselho Nacional de Desenvolvimento Científico e Tecnológico
CV: column volume
FAPESP: Fundação de AmparoàaàPesquisa do Estado de São Paulo
FICI: fractional inhibitory concentration index
GH19: glycoside hydrolase family 19
GH24: glycoside hydrolase family 24
IMAC: immobilized metal ion affinity chromatography
MBC: minimum bactericidal concentration
MDR: multidrug resistant
SEC: size exclusion chromatography
