# Supplementary material for: Combined effect of SAR-endolysin LysKpV475 with polymyxin B and Salmonella bacteriophage phSE-5
Source: Microbiology (Reading). 2024 May 13;170(5):001462. doi: 10.1099/mic.0.001462 (PMC11170124; doi:10.1099/mic.0.001462)
Supplement: Uncited Fig. S1. [file mic-170-01462-s002.pdf]

Supplementary figures

Combined effect of SAR-endolysin LysKpV475 with polymyxin B and *Salmonella* bacteriophage phSE-5

Marco Tulio Pardini Gontijo[1,2,†,*], Mateus Pereira Teles[1,3,4†], Hugo Martins Correia[1], Genesy Pérez Jorge[1,5], Isabella Carolina Rodrigues Santos Goes[1], Anthony Jhoao Fasabi Flores[1], Márcia Braz[4], Lucas de Moraes Ceseti[1], Priscila Zonzini Ramos[6], Ivan Rosa e Silva[3,7], Pedro Marcos Pereira Vidigal[8], Jörg Kobarg[7], Rafael Minguez Couñago[6,9], Cristina Elisa Alvarez-Martinez[1], Carla Pereira[4], Carmen Freire[10], Adelaide Almeida[4], Marcelo Brocchi[1]

[1] Departamento de Genética, Evolução, Microbiologia e Imunologia, Instituto de Biologia, Universidade Estadual de Campinas, Campinas, SP 13083-862, Brazil.

[2] Department of Molecular Genetics and Microbiology, Duke University School of Medicine, Durham, NC 27710, USA.

[3] Laboratório Nacional de Biociências (LNBio), Centro Nacional de Pesquisa em Energia e Materiais (CNPEM), Campinas, 13083-970 SP, Brazil.

[4] Department of Biology, and Centre for Environmental and Marine Studies (CESAM), University of Aveiro, Aveiro, Portugal.

[5] Departamento de Engenharia de Alimentos, Universidade de Córdoba, Monteria, Córdoba 230002, Colombia.

[6] Centro de Química Medicinal, Centro de Biologia Molecular e Engenharia Genética, Universidade Estadual de Campinas, Campinas, SP 13083-862, Brazil.

[7] Departamento de Ciências Farmacêuticas, Universidade Estadual de Campinas, Campinas, SP 13083-871, Brazil.

[8] Núcleo de Análise de Biomoléculas (NuBioMol), Universidade Federal de Viçosa (UFV), Viçosa, MG 36570-900, Brazil.

[9] Current Address: Structural Genomics Consortium and Division of Chemical Biology and Medicinal Chemistry, UNC Eshelman School of Pharmacy, University of North Carolina, Chapel Hill, North Carolina 27599, United States.

[10] Department of Chemistry, and Aveiro Institute of Materials (CICECO), University of Aveiro, Aveiro, Portugal.

[†] These authors contributed equally to this manuscript.

* Corresponding author

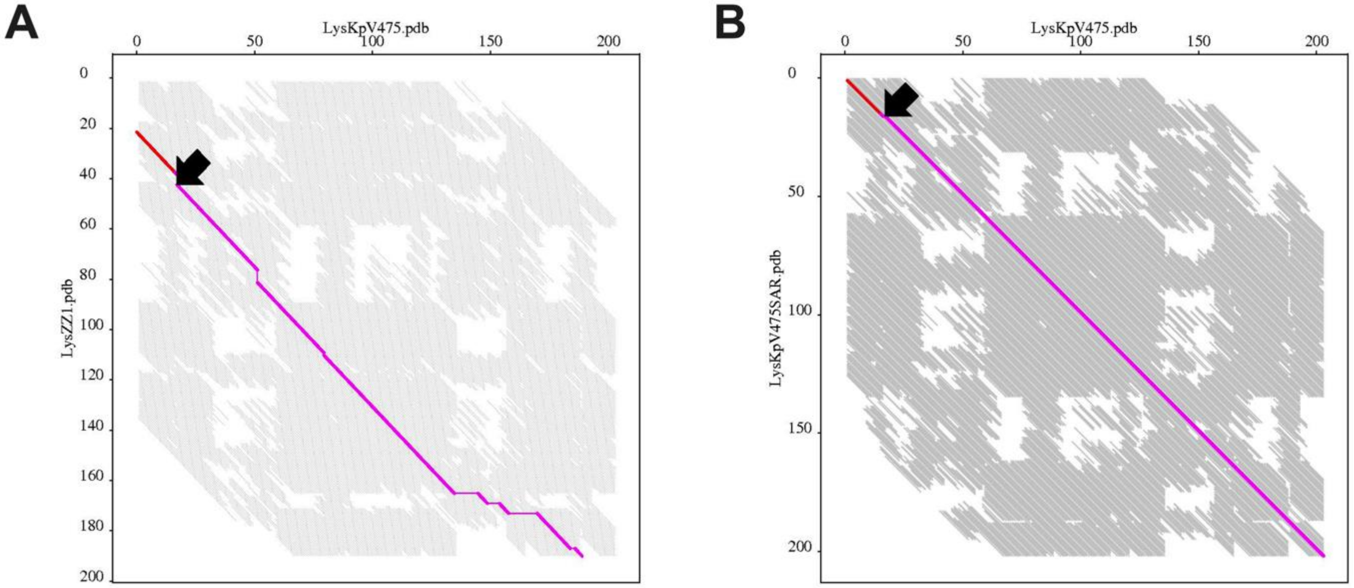

Fig. S1 Graph of FATCAT chaining results for the pairwise alignment of LysKpV475 vs. LysZZ1 (**A**) and LysKpV475 vs. LysKpV475ΔSAR (**B**). The arrow indicated a twist introduced at the hinge. A 45 degrees negative slope indicates a perfect alignment.

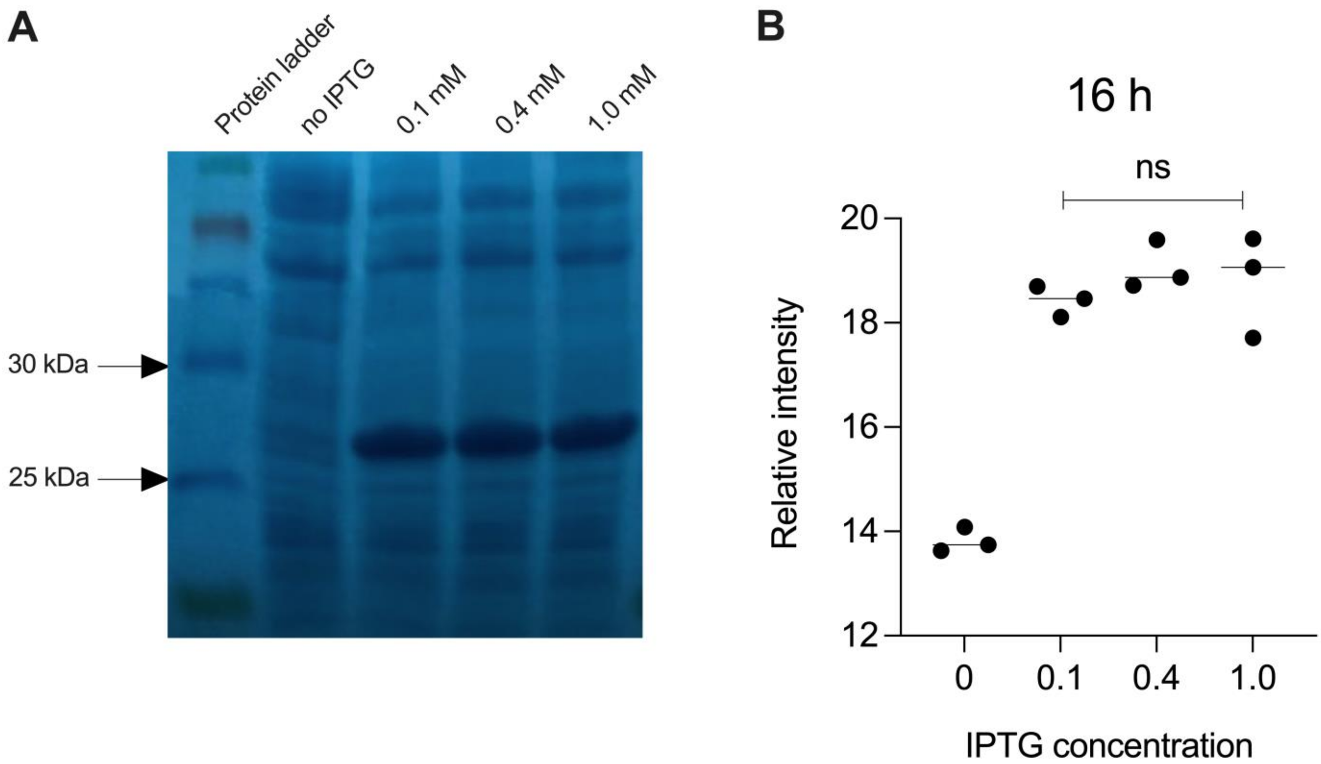

Fig. S2 SDS-PAGE results after protein induction (**A**) and the relative amount of LysKpV475 (approximately 22 kDa) 16 h after IPTG induction (**B**). The relative amount of LysKpV475 was quantified using Empiria Studio Software version 3.0 by comparing the intensity of the desired bands to the overall protein intensity loaded in the gel.

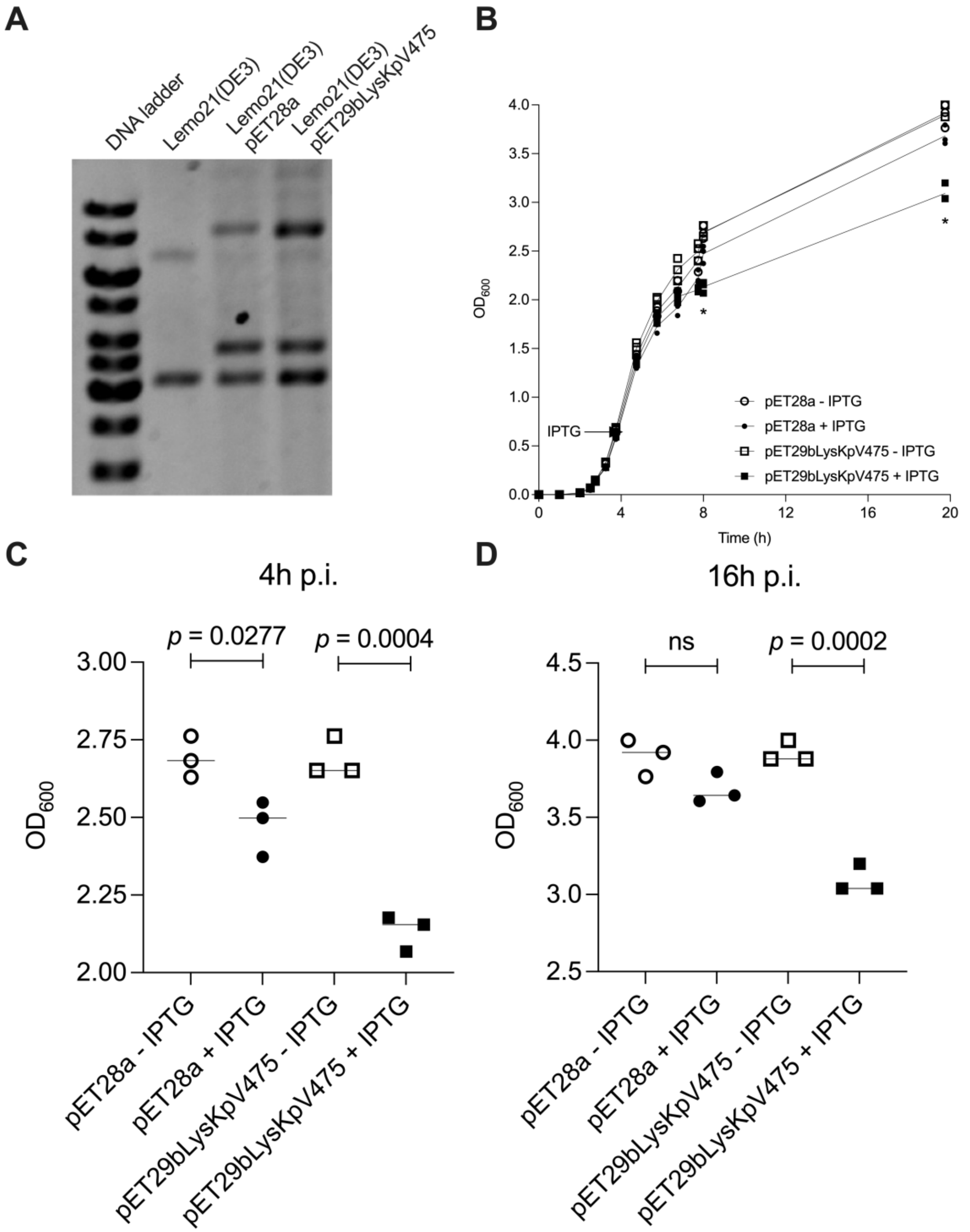

Fig. S3 *E. coli* Lemo21(DE3) transformed with plasmid pET28a and pET29b-LysKpV475. **A** Electrophoresis in 0.8% agarose gel for samples obtained from plasmid extraction. Lane M: 1 kb DNA marker; Lane 1: *E. coli* Lemo21(DE3); Lane 2: *E. coli* Lemo21(DE3) + pET28a; Lane 3: *E. coli* Lemo21(DE3) + pET29b-LysKpV475. **B** Growth curve of *E. coli* Lemo21(DE3) transformed with plasmid pET28a and pET19b-LysKpV475 induced and not induced with IPTG (0.1 mM0). The arrow indicates the point of induction with IPTG. Signs of protein expression are observed 4 (**C**) and 16 h (**D**) after induction.

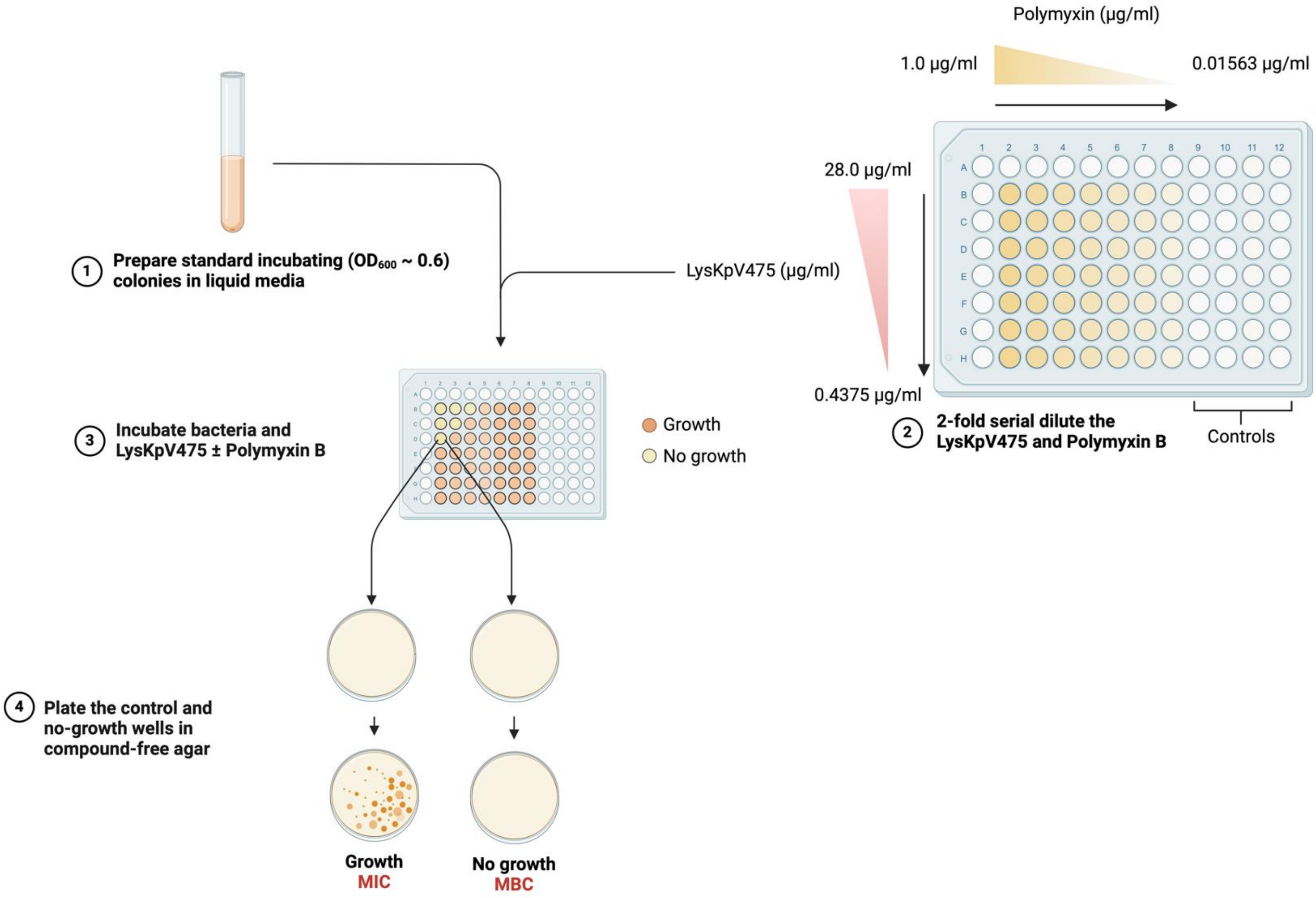

Fig. S4 Schematic representation of the checkerboard assay (CBA) utilized to determine the synergistic interactions between lyophilized LysKpV475 and outer membrane permeabilizer polymyxin B.
